# Return to Work Trajectories of Swedish Employees on Sick-Leave Due to Common Mental Disorders

**DOI:** 10.1007/s10926-024-10216-9

**Published:** 2024-06-22

**Authors:** Anna Toropova, Elisabeth Björk Brämberg, Gunnar Bergström

**Affiliations:** 1 Unit of Intervention and Implementation Research for Worker Health, Institute for Environmental Medicine, Karolinska Institute, 171 77 Stockholm, Sweden; 2 School of Public Health and Community Medicine, Institute of Medicine, University of Gothenburg, Gothenburg, Sweden; 3 Department of Occupational Health Sciences and Psychology, Faculty of Health and Occupational Studies, University of Gävle, Gävle, Sweden

**Keywords:** Common mental disorders, Return to work, Trajectories, Work environment

## Abstract

**Objectives:**

Recent research has emphasized that return to work (RTW) is a dynamic, gradual and often uneven process with a great degree of individual variation. This study aimed to identify RTW trajectories of Swedish employees on sick-leave due to common mental disorders (CMDs). The second aim was to explore which demographic, employment, health-related and work environment characteristics predicted RTW trajectory membership.

**Methods:**

Data comes from two 2-armed cluster-randomized controlled trials (RCT) with a 12-month follow-up. A participative problem-solving intervention aimed to reduce sick-leave was compared to care as usual (CAU) involving any kind of work-directed interventions. Participants on sick-leave due to CMDs at baseline (*N* = 197) formed the study sample. Latent growth mixture modeling and logistic regression were the main analytical approaches.

**Results:**

Five distinct RTW trajectories of Swedish employees were identified: Early RTW (*N* = 65), Delayed RTW (*N* = 50), Late RTW (*N* = 39), Struggling RTW (*N* = 21) and No RTW (*N* = 22). RTW trajectories differed consistently with regard to previous sick-leave duration and social support at work. More unique predictors of RTW trajectories included gender, rewards at work, work performance impairment due to health problems, home-to-work interference and stress-related exhaustion disorder.

**Conclusion:**

The study may have important clinical implications for identifying patients belonging to a particular RTW trajectory. Knowledge on the modifiable work environment factors that differentiated between the RTW trajectories could be useful for designing effective workplace interventions, tailored to particular needs of employees with CMDs. However, in a first step, the results need to be replicated.

**Supplementary Information:**

The online version contains supplementary material available at 10.1007/s10926-024-10216-9.

## Introduction

Common mental disorders (CMDs) are prevalent in the working population worldwide, with an yearly estimate of about 18% [1]. In this study, CMDs comprise anxiety, depression, adjustment and stress-related disorders. According to the recent report on the Global Burden of Diseases [2], mental disorders still account for the major disease burden, with no evident trend for reduction since the 1990s. In Sweden, they are the most common cause for sick-leave accounting for almost half of all registered sick-leave days, with women showing a higher risk for CMD-related sick-leave then men [3, 4]. CMDs have numerous negative effects on employees, workplaces and society at large. For an employee, the burden of CMDs includes individual suffering, impaired health, stigma as well as loss of income and social connections due to reduced or lost work ability [5–7]. The more detrimental consequences of long-term sick-leave due to CMDs include higher mortality risks due to cancer, cardiovascular diseases, and suicide [8]. For employers, CMD burden entails costs related to reduced productivity [9]. Finally, for the society, large healthcare and social security system costs are involved [10].

Considering the multiple burden of CMDs, the efforts facilitating full return to work (RTW) of employees on sick-leave due to CMDs are of utmost importance. A scoping review on the determinants of sickness absence and RTW in employees with CMDs [11] identified the following factors associated with return to work: age, the length of previous sick-leave, symptom severity, support from colleagues and managers and self-efficacy beliefs regarding RTW. A recent systematic review and meta-analysis on factors specifically predicting return to work for employees on sick-leave due to CMDs [12] found that return-to-work and general self-efficacy, favorable RTW expectations as well as better work ability were associated with return to work. However, predictors of RTW did not differ by diagnostic subgroups. Notably, both reviews emphasized the lack of knowledge on environmental, or work-related factors predicting RTW. Limited evidence concerns both employees’ perceptions of their work environment as well as objective worker and workplace characteristics, for example, type of work, employment conditions, working hours [12]. Previous research shows that organizational and social work environment factors, for example, social support, workload, psychological demands, reward and control over job tasks are important for employees’ mental health [13–15]. Given the need for more effective workplace interventions to facilitate RTW for employees with CMDs [11, 12, 16, 17] such knowledge is critical for developing interventions for this target group.

An important factor to consider in designing interventions towards RTW is that factors facilitating return to work at different time points may vary, calling for exploration of RTW predictors at specific time points. For example, in a study by Ekberg et al. [18] RTW within 3-month among employees sick-listed with CMDs was associated with a lower education level, more favorable treatment expectations, higher workability and lower perceptions of manager-employee justice. RTW occurring within 3 to 12 months was predicted by higher education level, higher need to reduce job demands and a stronger intention to leave the workplace [18].

However, several issues can be noted in relation to the plethora of previous research on RTW. First, the studies treat RTW as a stable characteristic, rather than a dynamic process [19]. Recent research shows that return to work is a process characterized by a great degree of individual variation, which is not addressed by the studies investigating time to RTW or RTW rates [20]. Moreover, RTW process is both gradual, where some employees return to work faster than others, as well as an uneven, where RTW trajectories for some employees involve more relapses than for others [17, 19, 21]. Finally, studies adopting a longitudinal perspective on individual RTW trajectories allow for investigation of sustainable RTW [22, 23]. Ultimately, knowledge on distinct RTW trajectories as well as potential predictors of these trajectories is important for the development of effective workplace interventions tailored to the employees belonging to a specific RTW trajectory.

## Aim

The first aim of this study was to identify RTW trajectories of employees on sick-leave due to CMDs. The follow-up period was 12 months after a workplace-directed intervention. The second aim was to explore which baseline factors with regard to demographic, employment, health-related and work environment characteristics predict RTW trajectory membership.

## Method

### Study Design and Setting

This study is based on data from two 2-armed cluster-randomized controlled trials (RCT) with a 12-month follow-up involving a participative problem-solving intervention for employees on sick-leave due to CMDs, compared to CAU involving any kind of work-directed interventions. One of the original studies, (registration number NCT02563743) [24] was carried out in three Swedish occupational health service units (*N* = 100 participants). Another study (registration number NCT3346395) [25] was conducted in cooperation with primary care centers (PCCs) in Västra Götland region of Sweden (*N* = 197 participants). A more detailed information about participant recruitment, randomization, the problem-solving intervention, can be found in the protocols for respective studies [24, 25]. Participants from both trials who were on sick-leave at baseline (*N* = 197) formed the sample for this study.

### Participants

For the study involving occupational health service units, participants were recruited between August 2015 and June 2017, while for the study with participation of primary care centers recruitment took place between February 2018 and February 2020. In the present study, the inclusion criteria were being on sick-leave due to CMDs (no longer than 3 months), approval of employer’s involvement and the ability to understand Swedish (written and spoken). Common exclusion criteria were severe mental illness, somatic workability-hindering disorders and pregnancy.

### Procedure

Data were collected by means of web-based surveys and registries. A web-based survey was administered at baseline, 6 and 12 months. Sick-leave registered data and diagnostic information was provided by the Swedish Social Insurance Agency (SSIA). For the purpose of this study, variables of interest common for both of the original datasets were merged into one.

### Measures

#### RTW Trajectory *Indicator*

The indicator of RTW trajectory was the number of register-based net sick-leave days (e.g. two days of 50% sickness absence were counted as one net day) per month throughout 12-month period. The range was 0 to 31 days. This data was complete for all participants. However, if a sickness absence spell was shorter than 14 days it was not registered by the SSIA.

Based on previous research, the following characteristics were used as potential predictors of RTW trajectory membership:

#### Demographic Characteristics

Demographic characteristics included gender and age.

#### Education and Employment Characteristics

Participants’ educational level referred to the highest level of education completed: compulsory school, upper secondary school/professional education, University education, postgraduate education. The question about work experience at the current workplace had five categories ‘less than 1 year’, ‘1–2 years’, ‘3–5 years’, ‘6–10 years’, ’more than 10 years’. The extent of overtime work was measured on a 6-point scale with responses ranging from 1 ‘Every day’ to 6 ‘Never’.

#### Mental Health and Stress-Related Symptoms

Self-rated anxiety and depression was assessed by 14-item Hospital Anxiety and Depression (HAD) scale [26], with 7 items measuring Anxiety and 7 items measuring depression. Responses ranged from 0 to 3, where higher scores mean higher levels of anxiety and depression. Stress-related exhaustion disorder was measured by the s-ED tool comprising three categories: non s-ED, moderate s-ED and pronounced s-ED [27].

#### Health Problems

A single-item question measured self-perceived general health, with responses ranging from 1 ‘Excellent’ to 5 ‘Poor’ [28]. Health-related quality of life (HRQoL) was assessed by the European Quality of Life questionnaire [29]. Sickness presenteeism was measured by a single-item question regarding the number of times being at work while sick during the past 12 months (1 ‘Never’ to 4 ‘More than 5 times’) [30]. Sleep quality was assessed with insomnia subscale of the Karolinska Sleep Questionnaire [31], where higher scores indicated better sleep quality. Previous sick-leave was measured with the number of net sick-leave days during the 12 months before baseline, retrieved from the Swedish Social Insurance Agency registry.

#### Work Ability

Three items from the Work Ability Index measured self-perceived work ability to meet physical and mental work demands (1- ‘Very good’ to 5- ‘Very poor’) as well 2-year work ability prognosis (1- ‘Probably not’ to 3- ‘Yes, quite sure’) [32]. The intention to return to work despite symptoms was measured on 1 to 5 scale, with higher scores denoting a stronger RTW intention [33]. Work performance impairment due to health and work environment problems was assessed by the items adapted from the Work Productivity Activity Impairment questionnaire—General Health Questionnaire (WPAI-GH) [34]. Responses ranged from 0 to 10, where higher scores indicated a greater work performance impairment.

#### Work Environment

Psychological demands, decision latitude and social support at work were assessed using the Swedish modified version of Demand-Control-Support Questionnaire [35, 36]. All dimensions were measured on a 4-point scale, with higher scores denoting higher psychological demands, more decision latitude and better social support at work.

Psychosocial factors in the work environment, such as perceived reward, fair and equal treatment from the manager, conflict between work tasks and employee’s values, as well as work-to-home and home-to-work interference were assessed with the General Nordic Questionnaire [37]. The items were assessed on a 5-point scale, with higher scores meaning higher perceived reward, fairer treatment from the manager, higher value conflict, and higher degree of work-to-home and home-to-work interference.

### Statistical Analysis

First, Latent Growth Mixture Model (LGMM) was used to identify participant subgroups following the same trajectories [38]. The LGMM model was estimated using maximum likelihood estimation. Since trajectory indicator represented longitudinal count-data (number of days), Poisson distribution was used. By means of modal assignment each subject was assigned the class to which they had the highest probability of belonging. The model was fitted for one to six classes with the help of ‘Traj’ plugin in the STATA statistical software [39].

The best class solution was decided on the basis of fit statistics indicating high probability of belonging to the most likely class. The following fit statistics were considered: log likelihood, Bayesian Information Criterion (BIC), Akaike Information Criterion (AIC) and entropy values. A model with the best fit would have the lowest values of log likelihood, BIC and AIC, while entropy value would be close to 1. An additional rule of thumb of the smallest class being not smaller than 5% of the total sample was followed [38]. The most appropriate model was determined by both fit indices values as well as theoretical soundness and clinical relevance of the trajectories.

Next, a detailed description of participant characteristics in each of the trajectories of the chosen model was presented. Finally, predictors of class membership from demographic, employment, health- and work-related characteristics were explored by means of binary logistic regression analysis, comparing trajectories pairwise. Analyses were performed with the help of SPSS 28 software. In a first step, univariable analyses with all baseline predictors was conducted for the initial selection of the variables which explain any variance in the outcome. In a second step, predictors with *p* < 0.1 were subsequently used in the multivariable analysis to achieve a more parsimonious model. In the multivariable analysis, a significance value of *p* < 0.05 was used to consider a predictor as statistically significant.

## Results

### Descriptive Statistics

Descriptive statistics for participants’ demographic, employment characteristics, and sick-leave status are presented in Table 1.

**Table 1:** Descriptive statistics of participant characteristics

Variable name	Total (*N* = 197)	Missing (*N*)
Age, *M* (SD)	43.4 (9.6)	0
Female, *N* (%)	167 (84.8)	0
Education level, *N* (%)		1
Compulsory school	5 (2.6)	
Upper secondary/professional education	92 (46.9)	
University/postgraduate education	99 (50.5)	
Work experience, years, *N* (%)		1
< 1	25 (12.8)	
1–2	44 (22.4)	
3–5	49 (25.0)	
6–10	34 (17.3)	
> 10	44 (22.4)	
Stress-related exhaustion disorderª, *N* (%)		4
No exhaustion disorder	21 (10.9)	
Moderate exhaustion disorder	27 (14.0)	
Pronounced exhaustion disorder	145 (75.1)	
Anxiety^b^, *N* (%)		0
Non-cases	34 (17.3)	
Possible cases	58 (29.4)	
Cases	105 (53.3)	
Depression^b^, *N* (%)		0
Non-cases	56 (28.4)	
Possible cases	61 (31.0)	
Cases	80 (40.6)	
Sick-leave rate, *N* (%)		1
25%	14 (7.1)	
50%	57 (29.1)	
75%	16 (8.2)	
100%	109 (55.6)	

ªStress-related exhaustion disorder (s-ED) [27]

^b^Hospital anxiety and depression (HAD) scale[26]

Women comprised the majority of the sample, with an average age of 43 years. A nearly equal share of the participants had either upper secondary or University education. A quarter of the sample reported 3 to 5 years of work experience, and somewhat smaller proportions worked for 1–2 or more than 10 years (22.4% in each category). The majority of the participants (75%) fell into ‘pronounced’ exhaustion syndrome category. Over half of the participants were classified as anxiety cases and 40% as depression cases. The majority of the participants were sick-listed at 100%, with the next largest category being sick-listed 50%.

### RTW Trajectories

Results of the LGMM for 6-class solution are demonstrated in Table 2.

**Table 2:** Fit statistics for models with one to six classes

Model	Log likelihood	AIC	BIC	Entropy	Proportion of the total sample in each class (%)
1 Class	− 5963.83	− 5965.83	− 5969.11	–	
2 Classes	− 4978.83	− 4984.83	− 4994.68	0.975	56/44
3 Classes	− 4966.93	− 4973.93	− 4985.42	0.966	57/32/11
4 Classes	− 4725.52	− 4736.52	− 4754.57	0.963	38/27/23/12
5 Classes	− 4607.34	− 4620.34	− 4641.68	0.960	33/26/19/11/11
6 Classes	− 4693.23	− 4707.23	8− 4730.21	0.968	43/2/27/12/9/7

It can be observed that with the addition of each next class until the 5-class solution, fit indices AIC, BIC and log likelihood as well as entropy values decreased. The 6-class solution was characterized by increased values of AIC, BIC and loglikelihood along with an increase in entropy. Despite having a somewhat higher entropy value compared to the 5-class solution, the 6-class model suggested only 2% of the sample in one of the classes, which was well below the minimum 5% requirement. Therefore, the final model with five classes was chosen based on the lowest loglikelihood, AIC and BIC values, as well as a reasonable and rather even distribution between classes. The final 5-class solution was also deemed theoretically sound and clinically relevant.

### RTW Trajectories Characteristics

**Fig. 1:**
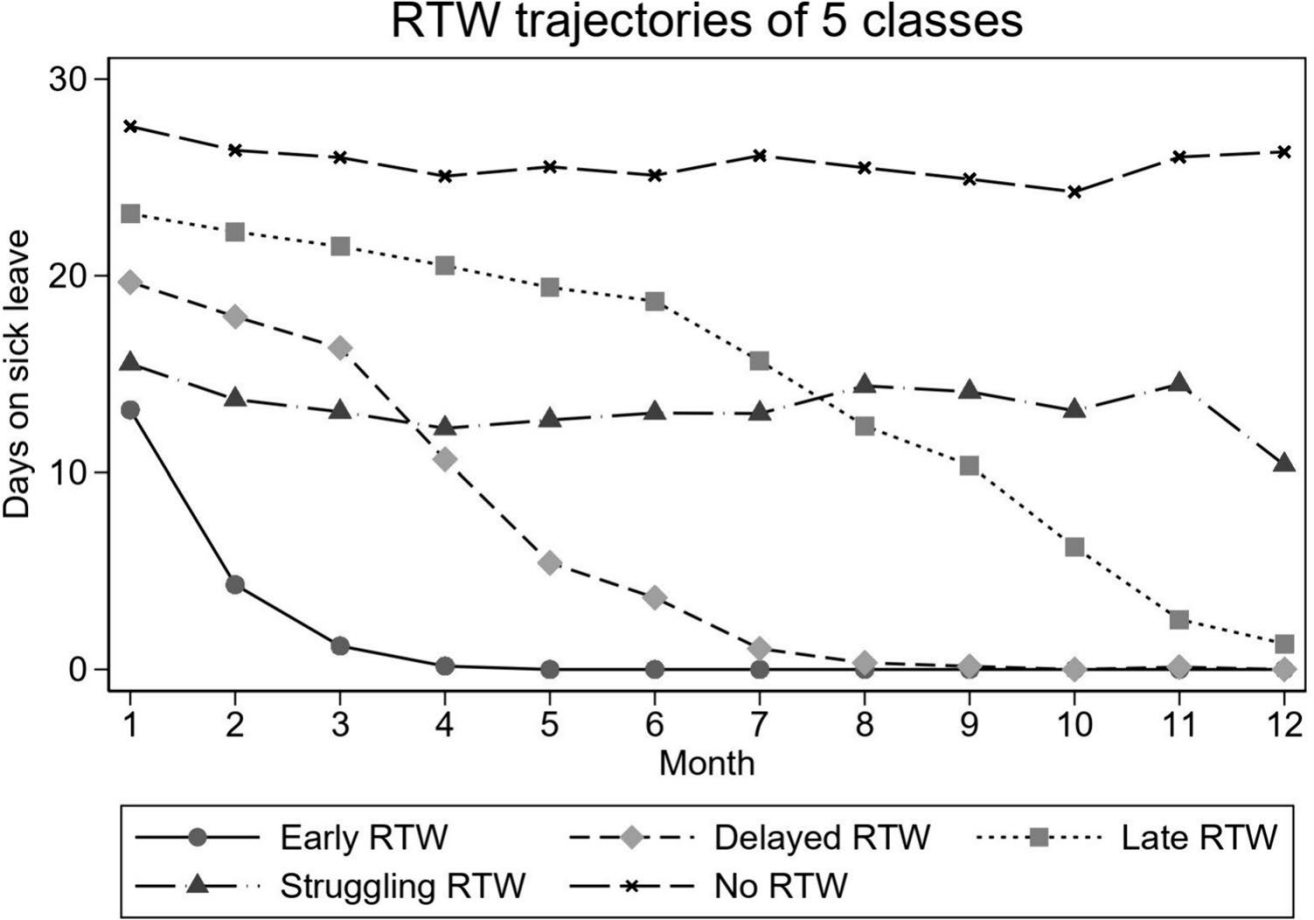
The 5-class RTW trajectories

The five-class RTW trajectories are presented in Fig. 1.

The largest trajectory class was labeled Early RTW, comprising nearly 33% of the total sample. Participants in the Early RTW class started with the lowest number of sick-leave days compared to other trajectories, have returned to and remained at work within 4 months. The next largest trajectory class, Delayed RTW (26% of the sample), started with more sick-leave days than the Early trajectory, and returned to work within 8 months. 19% of the participants belonged to the Late RTW trajectory, starting with more sick-leave days than the Delayed trajectory and having nearly reached RTW at 12 months. Participants in the Struggling RTW, comprising 11% of the total sample, have gone through periods of slowly decreasing sick-leave from 1 to 4 months to slowly increasing sick-leave from 4 to 8 months. Even though participants in the Struggling RTW trajectory experienced a steep decline in sick-leave from 11 to 12 months, they have still not returned to work at 12 months. Participants in this trajectory started with somewhat larger number of sick-leave days than the Early trajectory, but with quite fewer days compared to the Delayed trajectory. Finally, participants in No RTW trajectory comprised 11% of the sample. Similar to participants in the Struggling RTW trajectory, they have gone through periods of minor decreases and increases in sick-leave days, however unlike the Struggling trajectory, they experienced an increased sick-leave from 10 to 12 months. This group started with the highest amount of sick leave among the five trajectories and remained so throughout the 12-months period. Participant baseline characteristics within each of the five RTW trajectories are shown in Table 3.

**Table 3:** Participant characteristics within each of the five RTW trajectories

	Early RTW	Delayed RTW	Late RTW	Struggling RTW	No RTW	*p*
Baseline characteristics	*N* = 65	*N* = 50	*N* = 39	*N* = 21	*N* = 22	
Female, *n* (%)	49 (75.4)	42 (84.0)	35 (89.7)	20 (95.2)	20 (90.9)	0.380
Age, years, *m* (sd)	42.2 (9.6)	43.2 (9.8)	45.1 (8.2)	41.6 (11.7)	46.2 (9.0)	0.300
University education, *n* (%)	30 (46.2)	30 (60.0)	21 (53.8)	11 (52.4)	7 (31.8)	0.130
Work experience, *m* (sd)	2.8 (1.4)	3.1 (1.2)	3.4 (1.3)	3.3 (1.3)	3.6 (1.3)	0.098
Overtime work, *m* (sd), (1–6)	3.9 (1.8)	4.2 (1.5)	3.4 (1.6)	3.7 (1.6)	3.9 (1.8)	0.260
Anxiety, *m* (sd), (0–21)^a^	10.8 (4.7)	11.2 (3.8)	11.8 (4.0)	11.5 (3.5)	10.8 (4.7)	0.435
Depression, *m* (sd), (0–21)^a^	9.8 (4.1)	8.9 (4.1)	10.9 (4.2)	11.0 (3.8)	10.9 (3.5)	0.432
Exhaustion disorder, *m* (sd), (1–3)^b^	1.5 (0.7)	1.5 (0.8)	1.9 (0.3)	2.0 (0.2)	1.6 (0.7)	0.004
Self-reported health, *m* (sd), (1–5)^c^	3.4 (0.9)	3.5 (1.1)	3.6 (1.1)	4.0 (1.2)	3.9 (1.2)	0.160
Quality of life, *m* (sd), (0–100)^d^	69.7 (19.6)	61.2 (23.3)	53.9 (23.4)	56.4 (22.0)	56.7 (25.3)	0.005
Sleep quality, *m* (sd), (1–6)^e^	3.2 (1.4)	3.0 (1.3)	2.6 (1.1)	2.6 (1.2)	2.8 (1.3)	0.550
Sickness presenteeism, *m* (sd), (1–4)^f^	3.4 (0.9)	3.4 (0.8)	3.7 (0.7)	3.9 (0.4)	3.6 (0.6)	0.056
Previous sick-leave, days, *m* (sd), (1–31)	11.3 (7.5)	16.1 (9.6)	18.2 (8.9)	13.1 (6.2)	21.2 (10.4)	< 0.001
Impaired work ability_physical demands, *m* (sd), (1–5)^g^	2.4 (1.0)	2.7 (1.2)	3.2 (1.3)	2.8 (1.1)	3.2 (1.3)	0.003
Impaired work ability_mental demands, *m* (sd), (1–5)^g^	3.5 (0.9)	4.0 (0.9)	3.8 (0.8)	4.0 (0.8)	4.3 (0.8)	0.001
2-year work ability prognosis, *m* (sd), (1–3)^g^	2.4 (0.7)	2.4 (0.7)	2.2 (0.7)	2.5 (0.6)	2.0 (0.8)	0.072
RTW intention, *m* (sd), (1–5)^h^	3.6 (1.4)	2.8 (1.4)	2.9 (1.2)	3.2 (1.2)	2.8 (1.5)	0.026
Work impairment_health, *m* (sd), (0–10)^i^	6.0 (2.3)	6.5 (2.5)	8.1 (2.0)	6.2 (2.8)	6.9 (2.9)	0.001
Work impairment_work environement, *m* (sd), (0–10)^i^	5.9 (2.9)	6.0 (2.9)	7.5 (2.2)	6.2 (3.0)	6.9 (2.8)	0.038
Work demands, *m* (sd), (1–4)^j^	2.9 (0.7)	3.0 (0.7)	3.3 (0.6)	3.3 (0.5)	3.1 (0.5)	0.010
Work control, *m* (sd), (1–4)^j^	2.9 (0.5)	3.0 (0.5)	2.9 (0.4)	3.1 (0.4)	2.9 (0.5)	0.160
Social support at work, *m* (sd), (1–4)^j^	3.0 (0.5)	3.2 (0.5)	2.8 (0.4)	2.9 (0.6)	2.6 (0.6)	0.002
Value conflict, *m* (sd), (1–5)^k^	2.4 (1.1)	2.5 (1.2)	2.5 (1.2)	2.3 (1.0)	2.1 (1.2)	0.640
Reward at work, *m* (sd), (1–5)^k^	2.4 (1.0)	2.3 (1.2)	2.1 (1.1)	1.6 (0.7)	2.2 (1.1)	0.058
Fair treatment from manager, *m* (sd), (1–5)^k^	3.7 (1.2)	3.6 (1.1)	3.6 (1.1)	3.9 (1.3)	3.3 (1.4)	0.630
Work-to-Home Interference, *m* (sd), (1–5)^k^	3.3 (1.0)	3.4 (1.1)	3.8 (1.0)	3.6 (1.1)	3.7 (0.8)	0.240
Home-to-Work Interference, *m* (sd), (1–5)^k^	2.2 (1.1)	2.4 (1.3)	2.7 (1.2)	2.8 (1.2)	1.8 (0.8)	0.015
Diagnosis, *n* (% within class)	*N* = 57	*N* = 46	*N* = 31	*N* = 20	*N* = 15	< 0.001
Depression	11 (19.3)	15 (32.6)	3 (9.7)	7 (35.0)	2 (13.3)	
Anxiety	16 (28.1)	4 (8.7)	0 (0.0)	5 (25.0)	3 (20.0)	
Adjustment disorders	21 (36.8)	18 (39.1)	13 (41.9)	5 (25.0)	4 (26.7)	
Exhaustion syndrome	9 (15.8)	9 (19.6)	15 (48.4)	3 (15.0)	6 (40.0)	

^a^Hospital anxiety and depression (HAD) scale[26]

^b^Stress-related exhaustion disorder (s-ED)[27]

^c^Self-perceived general health[28]

^d^Health-related quality of life (HRQoL)[29]

^e^Insomnia subscale of the Karolinska Sleep Questionnaire[31]

^f^Number of times being at work while sick during the past 12 months[30]

^g^Work ability index (WAI)[32]

^h^The intention to return to work despite symptoms[33]

^i^Work productivity activity impairment questionnaire—General health questionnaire (WPAI-GH)[34]

^j^Demand-control-support questionnaire (DSCQ)[35]

^k^Nordic questionnaire for psychological and social factors at work—QPSNordic[37]

As can be seen from Table 3, the identified trajectories differed on the following characteristics: stress-related exhaustion disorder, quality of life, length of previous sick-leave, impaired work ability due to physical and mental demands at work, RTW intention, work impairment due to health- and work environment problems, work demands and social support at work as well as home-to-work interference. Below is the summary of the trajectory members’ scores on each of the above characteristics.

With regard to stress-related exhaustion disorder, the highest score was reported by the members of the *Struggling* RTW trajectory, while members of the *Early and Delayed* trajectories had the lowest scores. The highest quality of life was reported by the members of the *Early* RTW trajectory, with the *Late* trajectory members reporting the lowest quality of life. The longest previous sick-leave as well as the highest level of impaired work ability due to physical and mental demands at work characterized the members of *No RTW* trajectory. The highest level of impaired work ability due to physical demands at work was also reported by the members of the *Late* RTW trajectory. The shortest previous sick-leave and the lowest level of work impairment due to physical and mental demands at work characterized participants in the *Early* RTW trajectory.

The highest level of RTW intention despite symptoms was reported by the Early trajectory members, while the *Delayed and No* RTW trajectory members had the lowest levels. The highest scores on work impairment due to both health- and work environment problems were reported by the *Late* RTW trajectory members, while the *Early* RTW trajectory members had the lowest scores on both characteristics. The highest level of work demands characterized the members of the *Late and Struggling* RTW trajectories, while the Early trajectory members reported the lowest levels. The highest level of social support at work was reported by the *Delayed* RTW trajectory members, while *No* RTW trajectory members had the lowest levels of social support. Finally, the highest level of home-to-work interference characterized the *Struggling* RTW group members, while *No* RTW trajectory had the lowest level of home-to-work interference.

Results of univariable analysis can be found in Tables 1a–c of the Supplementary material. The univariable analysis showed significant differences between all of the RTW trajectories. In Tables 4, 5, and 6 results from a series of the multivariable analysis examining differences between the five RTW groups are presented. As can be observed in Tables 4, 5, and 6 most RTW trajectories differed on one or two predictors. Being a male and perceiving higher levels of reward at work decreased the odds of membership in the *Struggling* RTW compared to the *Early* RTW trajectory. A longer period of previous sick-leave increased the odds of membership in the *Delayed*, the *Late* and *No* RTW trajectories compared to the *Early* RTW trajectory. Higher levels of social support at work were associated with increased odds of the *Delayed* RTW group membership compared to being in the *Early* RTW group; as well as with decreased odds of the *Late* RTW membership compared to the *Delayed* RTW group. Higher levels of exhaustion disorder and more home-to-work interference decreased the odds of being in the *No* RTW group compared to the *Late* RTW. Finally, higher levels of work impairment due to health problems decreased the odds of belonging to the *Struggling* RTW trajectory compared to the *Late* RTW trajectory.

**Table 4:** Pairwise comparison of RTW trajectories—multivariable logistic regression

	Early (0), *N* = 65 vs. No RTW (1), *N* = 22		Delayed (0), *N* = 50 vs. No RTW (1), *N* = 22		Late (0), *N* = 39 vs. No RTW (1), *N* = 22	
	OR (95% CI)	*p*	OR (95% CI)	*p*	OR (95% CI)	*p*
Age	1.05 (0.97–1.15)	0.253				
Education level			0.41 (0.12–1.37)	0.148		
Work experience	1.49 (0.86–2.57)	0.151				
Depression			1.08 (0.92–1.25)	0.354		
Exhaustion disorder					0.14 (0.029–0.73)	**0.019**
Self-reported health	1.53 (0.77–3.04)	0.225				
Quality of life	0.97 (0.93–1.01)	0.085				
Previous sick-leave	1.17 (1.07–1.29)	** < 0.001**	1.04 (0.97–1.11)	0.262		
Work ability_physical demands	0.90 (0.47–1.71)	0.749				
Work ability_mental demands	2.14 (.79–5.77)	0.133			2.22 (0.95–5.17)	0.066
2-year RTW prognosis	0.72 (0.27–1.91)	0.507	1.16 (.48–2.82)	0.748		
RTW intention	0.79 (.49–1.29)	0.346				
Work impairment_health					0.74 (0.55–0.999)	0.050
Social support at work	0.65 (0.14–2.98)	0.583	0.30 (0.08–1.11)	0.071		
Home-to-work interference			0.72 (.40–1.31)	0.284	0.40 (0.18-0.88)	**0.022**

Values in bold are significant at 0.05 level

**Table 5:** Pairwise comparison of RTW trajectories—multivariable logistic regression

	Early (0), *N* = 65 vs. Struggling RTW (1), *N* = 21		Delayed (0), *N* = 50 vs. Struggling RTW (1), *N* = 21		Early (0), *N* = 65 vs. Late RTW (1), *N* = 39	
	OR (95% CI)	*p*	OR (95% CI)	*p*	OR (95% CI)	*p*
Gender	0.06 (0.003–0.97)	**0.047**				
Work experience					1.58 (0.99–2.54)	0.057
Depression			1.08 (0.92–1.27)	0.331		
Exhaustion disorder	7.27 (0.57–91.99)	0.126	5.45 (0.91–32.55)	0.063	3.07 (0.48–19.78)	0.238
Self-reported health	1.19 (0.52–2.75)	0.682				
Quality of life	0.98 (0.95–1.02)	0.299			0.98 (0.95–1.01)	0.255
Sleep quality					0.73 (0.43–1.24)	0.239
Presenteeism	1.62 (0.42–6.23)	0.483	2.51 (0.75–8.44)	0.137	1.01 (0.47–2.17)	0.980
Previous sick-leave					1.13 (1.04–1.22)	**0.004**
Work ability_physical demands	1.39 (0.57–3.42)	0.474			1.10 (0.52–2.33)	.805
Work ability_mental demands	1.33 (0.46–3.85)	0.597			0.73 (0.25–2.11)	0.556
Work impairment_health					1.46 (.97–2.19)	0.072
Work impairment_work environment					1.05 (0.79–1.40)	0.728
Work demands	5.28 (0.67–41.65)	0.114			2.28 (0.61–8.53)	0.220
Work control	5.72 (0.77–42.30)	0.088				
Social support at work			0.85 (0.27–2.70)	0.789	0.89 (0.19–4.27)	0.888
Value conflict						
Reward at work	0.33 (0.13–0.87)	**0.024**	0.52 (0.26–1.05)	0.069		
Work-to-home interference					1.06 (0.50–2.23)	0.883
Home-to-work interference	2.12 (0.90–4.98)	0.086			1.90 (0.98–3.66)	.056

Values in bold are significant at 0.05 level

^1^Female is coded as “1”, male is coded as “2”

**Table 6:** Pairwise comparison of RTW trajectories—multivariable logistic regression

	Early (0), *N* = 65 vs. Delayed RTW (1), *N* = 50		Delayed (0), *N* = 50 vs. Late RTW (1), *N* = 39		Late (0), *N* = 39 vs. Struggling RTW (1), *N* = 21		Struggling (0), *N* = 21 vs. No RTW (1), *N* = 22	
	OR (95% CI)	*p*	OR (95% CI)	*p*	OR (95% CI)	*p*	OR (95% CI)	*p*
Overtime work			0.79 (0.56–1.11)	0.173				
Depression			1.03 (0.90–1.19)	0.640				
Exhaustion disorder			2.94 (0.89–9.71)	0.078			0.46 (0.06–3.34)	0.441
Self-reported health								
Quality of life	0.98 (0.96–1.00)	0.118						
Presenteeism							0.45 (0.05–4.35)	0.490
Previous sick-leave	1.07 (1.01–1.13)	**0.017**			0.93 (0.86–1.01)	0.065	1.11 (0.99–1.25)	0.073
Work ability_physical demands	1.13 (0.73–1.75)	0.583	0.86 (0.53–1.41)	0.560				
Work ability_mental demands	1.43 (0.81–2.52)	0.223						
2-year RTW prognosis							0.21 (0.04–1.00)	0.050
RTW intention	0.77 (0.56–1.06)	0.105						
Work impairment_health			1.29 (0.97–1.71)	0.080	0.74 (0.56–0.99)	**0.039**		
Work impairment_work environment			1.02 (0.80–1.31)	0.877	0.96 (0.74–1.26)	0.773		
Work demands			0.98 (0.36–2.70)	0.971				
Work control					2.74 (0.57–13.20)	0.209		
Social support at work	3.41 (1.37–8.37)	**0.008**	0.26 (0.07–0.93)	**0.047**				
Reward at work							2.75 (0.86–8.79)	0.088
Home-to-work interference							0.45 (0.17–1.18)	0.103

Values in bold are significant at 0.05 level

Even though some differences between the trajectories were not statistically significant due to the small study sample, they may still bear clinical significance and are therefore worth noting. For instance, exhaustion disorder was associated with higher odds of the *Struggling* RTW group membership compared to the *Early* and the *Delayed* RTW trajectories; as well as with higher odds of being in the *Late* RTW trajectory compared to the *Early* and the *Delayed* RTW. Finally, the association between RTW trajectory membership and the experimental condition was examined, but was not statistically significant.

## Discussion

This study had two aims: to identify RTW trajectories of employees on sick-leave due to CMDs having received work directed interventions and to explore which baseline factors predict RTW trajectory membership. We found five distinct RTW trajectories. Three of the identified RTW trajectory classes have returned to work during the 12-month follow-up period, though at a different pace: Early, Delayed and Late RTW. Two RTW trajectory groups did not return to work and showed, despite some fluctuations, relatively stable courses of non-RTW over the follow-up period. These two trajectory classes differed regarding the level of sick-leave: No RTW group retained high sick-leave rates, whereas the Struggling trajectory group remained approximately on a half-time sick-leave level. The Early, Delayed, Late and No RTW trajectories were relatively similar to RTW trajectories found in earlier research [19–21].

Participants in the Early RTW trajectory had the lowest previous sick leave and scored high on RTW intention despite symptoms and quality of life at baseline. Most of the studies exploring heterogeneity of RTW process have identified a similar path of a relatively rapid and sustained return to work [19, 20, 23]. While these employees may need relatively limited interventions, possible adjustments at work to facilitate RTW appear reasonable. A steady and rather even RTW trajectory of the Delayed class resembled the ‘Recovery’ RTW trajectory identified by Sandin et al. [21]. Besides, in our study, this group reported a low RTW intention which may be a barrier for RTW [40], therefore potential reasons behind lack of RTW intention may be useful to address for these participants.

A characteristic that distinguished participants in the Late RTW trajectory from the Early and Delayed ones is that they started (month 1) with quite high levels of sick-leave but approached a full RTW after one year. This group may be prone to relapse, especially if their impaired work performance and lower work ability remains. These participants appear in need of work adjustments should their RTW be sustainable. The Late RTW trajectory in our study resembled a slow RTW with high relapse chance identified by Spronken et al. [19].

Further, previous research found trajectories similar to No RTW trajectory [20, 21], however in contrast to other studies, in our study, this group was the smallest. Participants in this trajectory may need more individually tailored support with their RTW process.

Members of the Struggling RTW trajectory started with a number of sick-leave days just above the Early trajectory, with their sick-leave fluctuating during the study period, making a steep turn towards return to work at month 12. A closer inspection of potential individual variations in the degrees of sick-leave among the strugglers indicated numerous relapses (see Table 2 in the Supplementary material). Moreover, the Struggling trajectory is illustrative of the complex and even paradoxical relationship between higher demands and increased presenteeism, described in the literature [41]. This group is also characterized by high control at work paired with low perception of reward at work.

Pairwise logistic regression analyses were conducted to explore which baseline factors with regard to demographic, education, employment, health-related and work environment characteristics predicted RTW trajectory membership. Within the demographic characteristics, gender was a significant predictor, while no statistically significant predictors were found in the education and employment category. Most of the predictors were within the work environment characteristics, while a few represented health/symptom-related factors. Among those, several predictors were consistent across trajectories.

For example, previous sick-leave of longer duration was associated with increased odds of being in a slower RTW trajectory (No RTW, Late and Delayed RTW compared to the Early RTW). This is in line with previous studies, which found that the duration of previous sick- leave predicted the length of future sickness absence [11, 13, 14]. Social support at work yielded less consistent results. Higher levels of support from the manager and colleagues were associated with decreased odds of being in the Late compared to the Delayed trajectory group, which is in agreement with the literature on positive effects of social support at work for faster RTW [11]. However, in case of the Early and Delayed RTW, higher support levels predicted membership in the latter trajectory group. This result underscores a more complex role of social support at work, both from the manager and colleagues, and the need for more tailored support, in terms of its kind and rigor, to participants following different RTW trajectories [23]. With regard to the Delayed RTW trajectory group, given their low RTW intention, social support could have been perceived as overly high expectations for their return to work.

We were also able to identify more unique characteristics of the distinct trajectories. For example, being a male and a perception of better reward at work was associated with decreased odds of being in the Struggling RTW trajectory compared to the Early RTW. The former finding is in line with previous studies which found that faster RTW trajectories had higher proportions of men [19, 21]. The perception of being rewarded at work was also linked to lower risks for sick-leave and faster return to work in previous research [15, 42]. Higher work performance impairment due to health problems was associated with decreased odds of being in the Struggling trajectory compared to the Late RTW. This underscores the need to ensure sustainable RTW for the Late RTW trajectory group. Higher levels of stress-related exhaustion disorder and home-to-work interference were associated with decreased odds of being in No RTW trajectory compared to the Late RTW trajectory. Employees with more pronounced levels of exhaustion disorder are also likely to have impaired work ability [27], which was characteristic of this trajectory group. While previous studies found the link between work-to-home interference and a longer time to RTW [43, 44], results of our study emphasize the need to additionally consider the role of home circumstances in the RTW process. In addition, when comparing different RTW trajectories, the impact of the social security system features on sick-leave of employees with CMDs, together with diagnoses and corresponding guidelines for sick-leave length, should also be considered [23].

A number of the identified RTW trajectory predictors, despite failing to reach statistical significance, may still be clinically relevant and deserve further examination. For example, exhaustion disorder stood out as a consistent predictor of later RTW trajectories, with apparent clinical significance for identifying employees at risk for prolonged periods of sick-leave and designing tailored interventions to facilitate their return to work. The prevalence of exhaustion syndrome diagnosis in the Late and No RTW trajectories provides further support for clinical relevance of these results.

Provided that the trajectory classes described above are replicable and clinically meaningful, two overarching challenges arise: first, how to identify these groups early in the sick-leave process and second, how to customize interventions to meet the potentially different needs of each of the trajectory classes. For instance, employees in the Early and Delayed RTW trajectories may benefit from shorter workplace-directed intervention, whereas those in the Late RTW may require more support concerning their working conditions and/or work environment. In case of the Struggling trajectory, characterized by multiple sick-leave relapses, continuous treatment combined with workplace adaptations would most probably be beneficial. To the best of the authors’ knowledge, evaluations of tailored workplace interventions among employees with CMDs, based on valid measurement methods including both health and workplace factors, are currently lacking.

## Methodological Considerations

Our study has several limitations. First, the sample was relatively small in relation to analysis complexity, potentially compromising the study’s statistical power and leading to false-negative findings. Next, this study was exploratory using the analytical approach allowing to identify multiple trajectories, however the focus was also on their theoretical, empirical and clinical validity. Furthermore, a large number of statistical tests were conducted increasing the risk of type I error. Some caution is needed when comparing the identified RTW trajectories with the ones found in other studies. In particular, differences in the study populations should be considered even if the participants have diagnosis included in CMDs. For example, Arends et al. study [23] only included employees prepared for RTW, while Hellström et al. [20] study population was recently diagnosed with affective and anxiety disorders, which are not fully comparable with the present study’s population.

One of the main strengths of our study is that RTW trajectory indicator was based on register data, ensuring no loss to follow-up as well as higher accuracy in identifying participant trajectories. Further, to the best of our knowledge, this study used one of the most comprehensive set of predictors of trajectory membership covering participant demographic, health-related, work ability and work environment factors relevant for distinguishing between different RTW pathways. Moreover, all of our measures were validated instruments. A particular strength of this study is inclusion of work environment factors identified in previous studies as important for studying heterogeneity in RTW process but lacking in current research.

Further research would benefit from a replication study in a larger sample to confirm the existence of the identified trajectories, in particular with regard to the Struggling trajectory as it stands out in relation to previous research on RTW heterogeneity. Besides, a longer follow-up period to monitor whether participants in the identified RTW trajectories have sustained their return to work is warranted.

## Concluding Remarks

This study found five distinct RTW trajectories of Swedish employees on sick-leave due to common mental disorders, which differed with regard to participant health-related and work environment characteristics. These results may have clinical implications for identifying patients belonging to a particular RTW trajectory. As identified RTW trajectories also differed on multiple modifiable work environment factors, this knowledge can be useful for designing effective workplace interventions, tailored to particular needs of employees with CMDs.

## Supplementary Information

Below is the link to the electronic supplementary material.

Supplementary file1 (DOCX 41 kb)
