# Supplementary material for: Return to Work Trajectories of Swedish Employees on Sick-Leave Due to Common Mental Disorders
Source: J Occup Rehabil. 2024 Jun 22;35(3):479–90. doi: 10.1007/s10926-024-10216-9 (PMC12361341; doi:10.1007/s10926-024-10216-9)
Supplement: Supplementary file 1 — Supplementary file1 (DOCX 41 kb) [file 10926_2024_10216_MOESM1_ESM.docx]

# **Table 1a. Pairwise comparison of RTW trajectories – univariable logistic regression**

|  | **Early (0), N=65 vs. No RTW (1), N=22** | | **Delayed (0), N=50 vs. No RTW (1), N=22** | | **Late (0), N=39 vs. No RTW (1), N=22** | |
| --- | --- | --- | --- | --- | --- | --- |
|  | OR (95% CI) | *p* | OR (95% CI) | *p* | OR (95% CI) | *p* |
| Gender | .33 (.07-1.56) | .161 | .53 (.10-2.70) | .441 | .88 (.15-5.21) | .883 |
| Age | 1.05 (.99-1.11) | **.092** | 1.04 (.98-1.09) | .224 | 1.02 (.95-1.08) | .638 |
| Education level | .57 (.22-1.45) | .238 | .38 (.15-.99) | **.047** | .48 (.18-1.27) | .140 |
| Work experience | 1.53 (1.05-2.21) | **.025** | 1.38 (.90-2.13) | .145 | 1.17 (.77-1.76) | .465 |
| Overtime work | 1.01 (.77-1.33) | .938 | .89 (.65-1.21) | .444 | 1.20 (.87-1.67) | .272 |
| Anxiety | 1.00 (.90-1.11) | .955 | .98 (.86-1.11) | .741 | .94 (.83-1.07) | .375 |
| Depression | 1.08 (.95-1.22) | .238 | 1.14 (.998-1.30) | **.053** | 1.00 (.88-1.15) | .962 |
| Exhaustion disorder | 1.07 (.53-2.16) | .853 | 1.17 (.60-2.28) | .655 | .26 (.07-.89) | **.033** |
| Self-reported health | 1.78 (1.02-3.13) | **.043** | 1.45 (.88-2.37) | .143 | 1.30 (.80-2.13) | .289 |
| Quality of life | .98 (.95-1.00) | **.020** | .99 (.97-1.01) | .467 | 1.01 (.98-1.03) | .657 |
| Sleep quality | .79 (.55-1.14) | .212 | .88 (.60-1.30) | .517 | 1.14 (.74-1.79) | .538 |
| Presenteeism | 1.46 (.74-2.85) | .273 | 1.41 (.66-2.98) | .375 | .83 (.37-1.87) | .652 |
| Previous sick-leave | 1.14 (1.07-1.22) | **<.001** | 1.06 (1.00-1.12) | **.052** | 1.04 (.98-1.10) | .235 |
| Work ability_physical demands | 1.96 (1.24-3.08) | **.004** | 1.41 (.92-2.18) | .117 | 1.03 (.68-1.57) | .886 |
| Work ability_mental demands | 3.10 (1.55-6.22) | **.001** | 1.61 (.83-3.12) | .156 | 1.97 (.95-4.07) | **.069** |
| 2-year RTW prognosis | .46 (.24-.88) | **.019** | .51 (.26-1.00) | **.049** | .62 (.30-1.25) | .182 |
| RTW intention | .69 (.48-.98) | **.040** | .99 (.70-1.42) | .978 | .94 (.63-1.40) | .750 |
| Work impairment_health | 1.17 (.94-1.46) | .167 | 1.06 (.87-1.29) | .568 | .80 (.64-1.02) | **.069** |
| Work impairment_work environment | 1.14 (.95-1.37) | .166 | 1.12 (.93-1.35) | .245 | .90 (.72-1.11) | .316 |
| Work demands | 1.72 (.75-3.99) | .204 | 1.21 (.54-2.71) | .650 | .49 (.18-1.29) | .147 |
| Work control | 1.01 (.36-2.86) | .985 | .53 (.18-1.52) | .234 | .90 (.26-3.11) | .869 |
| Social support at work | .35 (.14-.87) | **.023** | .19 (.07-.55) | **.002** | .56 (.20-1.55) | .260 |
| Value conflict | .79 (.49-1.26) | .315 | .75 (.49-1.17) | .209 | .74 (.47-1.19) | .216 |
| Reward at work | .81 (.49-1.33) | .393 | .94 (.60-1.47) | .786 | 1.11 (.69-1.79) | .656 |
| Fair treatment_manager | .80 (.54-1.17) | .239 | .80 (.52-1.22) | .294 | .85 (.55-1.32) | .469 |
| Work-to-home interference | 1.52 (.87-2.66) | .145 | 1.29 (.76-2.21) | .348 | .90 (.51-1.59) | .722 |
| Home-to-work interference | .67 (.40-1.10) | .111 | .61 (.37-1.01) | **.052** | .40 (.22-.75) | **.004** |

**Table 1b. Pairwise comparison of RTW trajectories – univariable logistic regression**

|  | **Early (0), N=65 vs. Struggling RTW (1), N=21** | | **Delayed (0), N=50 vs. Struggling RTW (1), N=21** | | **Early (0), N=65 vs. Late RTW (1), N=39** | |
| --- | --- | --- | --- | --- | --- | --- |
|  | OR (95% CI) | *p* | OR (95% CI) | *p* | OR (95% CI) | *p* |
| Gender^1^ | .16 (.20-1.32) | **.089** | .26 (.03-2.24) | .222 | .37 (.11-1.22) | .103 |
| Age | .995 (.95-1.05) | .846 | .99 (.94-1.04) | .572 | 1.04 (.99-1.09) | .112 |
| Education level | 1.28 (.53-3.07) | .584 | .85 (.35-2.04) | .707 | 1.26 (.60-2.66) | .540 |
| Work experience | 1.28 (.89-1.83) | .178 | 1.10 (.73-1.66) | .654 | 1.33 (.99-1.79) | **.056** |
| Overtime work | .93 (.70-1.24) | .614 | .79 (.56-1.10) | .164 | .86 (.68-1.09) | .202 |
| Anxiety | 1.04 (.93-1.17) | .483 | 1.03 (.89-1.18) | .702 | 1.06 (.96-1.16) | .256 |
| Depression | 1.08 (.95-1.22) | .238 | 1.13 (.996-1.29) | **.057** | 1.07 (.97-1.17) | .205 |
| Exhaustion disorder | 7.24 (1.04-50.58) | **.046** | 5.60 (.95-33.07) | **.058** | 4.76 (1.49-15.25) | **.009** |
| Self-reported health | 1.83 (1.04-3.20) | **.036** | 1.48 (.90-2.42) | .123 | 1.21 (.80-1.83) | .377 |
| Quality of life | .97 (.95-.99) | **.014** | .99 (.97-1.01) | .420 | .97 (.95-.99) | **<.001** |
| Sleep quality | .73 (.49-1.08) | .112 | .80 (.52-1.22) | .298 | .69 (.50-.96) | **.026** |
| Presenteeism | 3.60 (1.11-11.65) | **.033** | 4.14 (1.15-14.90) | **.030** | 1.67 (.94-2.95) | **.079** |
| Previous sick-leave | 1.04 (.97-1.11) | .314 | .96 (.90-1.02) | .194 | 1.11 (1.05-1.17) | **<.001** |
| Work ability_physical demands | 1.50 (.93-2.39) | **.094** | 1.06 (.67-1.67) | .799 | 1.90 (1.30-2.77) | **<.001** |
| Work ability_mental demands | 2.04 (1.10-3.81) | **.025** | 1.06 (.57-1.95) | .855 | 1.65 (1.02-2.66) | **.042** |
| 2-year RTW prognosis | 1.27 (.60-2.66) | .534 | 1.42 (.66-3.04) | .374 | .71 (.41-1.22) | .215 |
| RTW intention | .81 (.56-1.17) | .265 | 1.22 (.84-1.79) | .300 | .70 (.52-.95) | .021 |
| Work impairment_health | 1.03 (.84-1.27) | .770 | .96 (.79-1.17) | .665 | 1.69 (1.30-2.21) | **<.001** |
| Work impairment_work environment | 1.04 (.88-1.24) | .651 | 1.02 (.86-1.22) | .801 | 1.29 (1.08-1.54) | **.005** |
| Work demands | 3.07 (1.19-7.94) | **.021** | 2.03 (.82-5.03) | .128 | 3.04 (1.46-6.34) | **.003** |
| Work control | 3.56 (1.00-12.68) | **.051** | 1.52 (.48-4.79) | .477 | 1.11 (.45-2.72) | .820 |
| Social support at work | .74 (.31-1.78) | .501 | .42 (.16-1.07) | **.069** | .50 (.22-1.12) | **.092** |
| Value conflict | .89 (.55-1.43) | .619 | .83 (.53-1.29) | .401 | 1.11 (.77-1.59) | .581 |
| Reward at work | .36 (.18-.71) | **.003** | .53 (.30-.94) | **.030** | .73 (.49-1.08) | .115 |
| Fair treatment_manager | 1.12 (.74-1.70) | .581 | 1.19 (.75-1.89) | .467 | .91 (.64-1.28) | .574 |
| Work-to-home interference | 1.34 (.80-2.26) | .269 | 1.18 (.72-1.93) | .523 | 1.59 (1.02-2.46) | **.040** |
| Home-to-work interference | 1.56 (1.01-2.40) | **.044** | 1.30 (.87-1.94) | .200 | 1.42 (1.00-2.02) | **.050** |

^1^Female is coded as “1”, male is coded as “2”

**Table 1c. Pairwise comparison of RTW trajectories – univariable logistic regression**

|  | **Early (0), N=65 vs. Delayed RTW (1), N=50** | | **Delayed (0), N=50 vs. Late RTW (1), N=39** | | **Late (0), N=39 vs. Struggling RTW (1), N=21** |  | **Struggling (0), N=21 vs. No RTW (1), N=22** |  |
| --- | --- | --- | --- | --- | --- | --- | --- | --- |
|  | OR (95% CI) | *p* | OR (95% CI) | *p* | OR (95% CI) | *p* | OR (95% CI) | *p* |
| Gender | .62 (.24-1.61) | .329 | .60 (.17-2.16) | .435 | .44 (.05-4.19) | .473 | 2.00 (.17-23.86) | .584 |
| Age | 1.01 (.97-1.05) | .574 | 1.02 (.98-1.07) | .319 | .96 (.91-1.02) | .184 | 1.04 (.98-1.11) | .161 |
| Education level | 1.61 (.80-3.26) | .184 | .80 (.37-1.72) | .561 | 1.03 (.42-2.53) | .945 | .53 (.19-1.47) | .220 |
| Work experience | 1.21 (.91-1.60) | .194 | 1.15 (.82-1.60) | .422 | .96 (.64-1.43) | .838 | 1.22 (.76-1.97) | .407 |
| Overtime work | 1.13 (.91-1.41) | .279 | .72 (.54-.95) | **.021** | 1.10 (.774-1.56) | .595 | 1.10 (.76-1.60) | .610 |
| Anxiety | 1.02 (.94-1.12) | .613 | 1.05 (.94-1.17) | .415 | .98 (.85-1.13) | .770 | .96 (.83-1.11) | .572 |
| Depression | .95 (.87-1.04) | .258 | 1.12 (1.01-1.24) | **.041** | 1.01 (.88-1.15) | .932 | .997 (.84-1.18) | .968 |
| Exhaustion disorder | .90 (.55-1.47) | .679 | 4.07 (1.39-11.93) | **.010** | 1.68 (.16-17.26) | .664 | .18 (.03-1.29) | **.088** |
| Self-reported health | 1.07 (.73-1.56) | .726 | 1.10 (.75-1.61) | .642 | 1.33 (.82-2.16) | .251 | .97 (.58-1.62) | .903 |
| Quality of life | .98 (.97-1.00) | **.039** | .987 (.97-1.01) | **.**150 | 1.01 (.98-1.03) | .688 | 1.00 (.98-1.03) | .959 |
| Sleep quality | .89 (.68-1.17) | .407 | .76 (.54-1.08) | .130 | 1.05 (.65-1.70) | .848 | 1.10 (.67-1.81) | .717 |
| Presenteeism | 1.09 (.70-1.67) | .710 | 1.62 (.86-3.06) | .135 | 2.10 (.61-7.21) | .239 | .30 (.07-1.23) | **.095** |
| Previous sick-leave | 1.07 (1.02-1.12) | **.004** | 1.02 (.98-1.07) | .300 | .92 (.86-.99) | **.031** | 1.11 (1.03-1.21) | **.009** |
| Work ability_physical demands | 1.38 (.98-1.96) | **.069** | 1.37 (.95-1.97) | **.089** | .77 (.50-1.21) | .260 | 1.34 (.80-2.23) | .263 |
| Work ability_mental demands | 1.89 (1.21-2.98) | **.006** | .85 (.52-1.40) | .528 | 1.26 (.65-2.43) | .496 | 1.51 (.71-3.22) | .284 |
| 2-year RTW prognosis | .902 (.55-1.49) | .687 | .79 (.45-1.39) | .415 | 1.90 (.83-4.36) | .128 | .33 (.13-.83) | **.019** |
| RTW intention | .69 (.53-.90) | **.007** | 1.06 (.78-1.45) | .719 | 1.20 (.77-1.87) | .419 | .81 (.51-1.28) | .362 |
| Work impairment_health | 1.09 (.93-1.27) | .303 | 1.38 (1.11-1.71) | **.004** | .70 (.55-.92) | **.009** | 1.09 (.88-1.36) | .432 |
| Work impairment_work environment | 1.02 (.90-1.16) | .797 | 1.26 (1.05-1.52) | **.012** | .81 (.65-1.01) | **.064** | 1.09 (.88-1.35) | .439 |
| Work demands | 1.32 (.75-2.30) | .332 | 2.08 (1.02-4.23) | **.044** | .90 (.33-2.41) | .829 | .44 (.12-1.63) | .216 |
| Work control | 1.95 (.87-4.37) | .103 | .53 (.20-1.37) | .187 | 4.09 (.92-18.27) | **.065** | .27 (.06-1.31) | .105 |
| Social support at work | 1.93 (.92-4.04) | **.082** | .22 (.08-.58) | **.002** | 1.38 (.47-4.01) | .557 | .53 (.20-1.46) | .219 |
| Value conflict | 1.10 (.80-1.53) | .558 | .999 (.71-1.42) | .995 | .82 (.50-1.32) | .405 | .88 (.50-1.56) | .662 |
| Reward at work | .89 (.63-1.26) | .103 | .85 (.59-1.23) | .398 | .64 (.36-1.14) | .128 | 2.05 (.98-4.32) | **.058** |
| Fair treatment_manager | .97 (.70-1.33) | .828 | .93 (.63-1.37) | .710 | 1.27 (.78-2.05) | .337 | .73 (.46-1.17) | .192 |
| Work-to-home interference | 1.10 (.77-1.58) | .599 | 1.36 (.89-2.08) | .151 | .87 (.52-1.45) | .587 | 1.08 (.56-2.08) | .822 |
| Home-to-work interference | 1.13 (.83-1.53) | .431 | 1.21 (.86-1.70) | .280 | 1.11 (.70-1.76) | .648 | .36 (.18-.74) | **.005** |

**Table 2.** Percentage sick-leave^a^ of the Struggling trajectory (0 – no sick-leave)

| **ID** | **m1** | **m2** | **m3** | **m4** | **m5** | **m6** | **m7** | **m8** | **m9** | **m10** | **m11** | **m12** |
| --- | --- | --- | --- | --- | --- | --- | --- | --- | --- | --- | --- | --- |
| 1 | 50 | 50 | 75 | 75 | 75 | 75 | 75 | 75 | 75 | 100 | 75 | 75 |
| 2 | 100 | 75 | 100 | 25 | 0 | 0 | 0 | 0 | 0 | 0 | 0 | 75 |
| 3 | 75 | 75 | 25 | 75 | 75 | 100 | 100 | 100 | 100 | 25 | 100 | - |
| 4 | 100 | 100 | 100 | 0 | 0 | 0 | 25 | 50 | 0 | 0 | 0 | 0 |
| 5 | 75 | 75 | 75 | 75 | 100 | 100 | 100 | 100 | 100 | 100 | 100 | 0 |
| 6 | 25 | 25 | 25 | 25 | 50 | 75 | 75 | 75 | 75 | 75 | 100 | 100 |
| 7 | 50 | 50 | 50 | 50 | 50 | 50 | 75 | 75 | 75 | 75 | 75 | 75 |
| 8 | 25 | 50 | 75 | 75 | 75 | 75 | 75 | 75 | 25 | 25 | 75 | 100 |
| 9 | 75 | 75 | 75 | 75 | 75 | - | 50 | 100 | 100 | 100 | - | - |
| 10 | 100 | 0 | 0 | 0 | 0 | 0 | 0 | 0 | 0 | 25 | 25 | 0 |
| 11 | 75 | 75 | 100 | 100 | 100 | 25 | 100 | 100 | 100 | 25 | 0 | 100 |
| 12 | 25 | 50 | 100 | 75 | 75 | 100 | 100 | 100 | 100 | 100 | 0 | 0 |
| 13 | 50 | 100 | 75 | 75 | 75 | 75 | 75 | 75 | 25 | 100 | 100 | 100 |
| 14 | 75 | 75 | 75 | 75 | - | 75 | - | - | - | - | - | - |
| 15 | 75 | 75 | 75 | 25 | 100 | 100 | 100 | - | - | 100 | 100 | - |
| 16 | 75 | 75 | 75 | 100 | 100 | 100 | 100 | 100 | 100 | 100 | 100 | 25 |
| 17 | 25 | 0 | 0 | 0 | 75 | 75 | 75 | 75 | 100 | 25 | 100 | 100 |
| 18 | 0 | 0 | 25 | 75 | 75 | 25 | 25 | 25 | 25 | 25 | 25 | 50 |

^a^ Data comes from participant self-reported text-message survey (data missing for 3 participants)
